# Enhancing employee well-being through a culturally adapted training program: a mixed-methods study in South Africa

**DOI:** 10.3389/fpubh.2025.1627464

**Published:** 2025-08-18

**Authors:** Anurag Shekhar, Musawenkosi D. Saurombe, Renjini Mary Joseph

**Affiliations:** Department of Industrial Psychology and People Management, College of Business and Economics, University of Johannesburg, Johannesburg, South Africa

**Keywords:** employee well-being intervention, mental health, human resource management, cultural adaptation, South African workplace

## Abstract

**Introduction:**

Structured, well-being interventions are under-researched in non-Western workplaces. This study evaluates *The Good Life* training program—a participatory, multi-component training intervention—on employee well-being, engagement and stress in South Africa.

**Methods:**

Employing an exploratory, quasi-experimental, explanatory sequential mixed-methods design, we collected quantitative data from 50 South African respondents across three delivery formats (four half-days online, two full-days in classroom, and four half-days in classroom) at pre-training and 3 months post-training using five validated scales (PSS-4, UWES-3, SWLS, FS, WEMWBS-14). No concurrent control group was retained due to attrition and contamination; thus, causal inferences are cautious. Qualitative data were gathered via semi-structured interviews with a purposive subsample of 15 participants to elucidate mechanisms of change.

**Results:**

Two full-day workshops led to significant improvements in overall well-being and work engagement, whereas the online format produced a significant boost in well-being only. The half-day format showed no statistically significant changes. Qualitative findings highlighted immersive peer interaction, structured reflection and managerial support as core drivers of impact.

**Discussion:**

Immersive, HR-facilitated training shows promise for enhancing well-being and engagement in South African workplaces. Future research should employ randomized controlled designs, larger samples and objective measures (e.g., absenteeism, physiological indicators) to substantiate and extend these preliminary findings.

## 1 Introduction

The COVID-19 pandemic shone a stark light on a mounting mental health emergency that long predated the surge and associated implications of the coronavirus (1). Even before 2020, the prevalence of depression, anxiety and other disorders was rising globally (2), with young people particularly at risk as many such conditions begin to amplify during adolescence and early adulthood (3). At the same time, the chronic shortage of mental-health professionals—and the failure of massive investments in treatment and research to stem the tide of illness—has revealed the limitations of a care model built almost exclusively around diagnosis and therapy (2, 4, 5). Despite high per-capita spending and an abundance of specialists in wealthier nations, population-level indicators continue to deteriorate, underscoring the need for a radically different approach (2).

Public health disciplines have repeatedly called for such a reimagining of mental-health policy and practice. Major reports—including successive Lancet Commissions (6), World Health Organization reviews (7–9) and global disease-control initiatives—urge a shift away from focusing solely on treating established illnesses toward preventing mental-health problems and actively promoting psychological well-being (2). This first principle recognizes that, even under optimal circumstances, treatment alone can only reduce the global burden of depression by about one-third, and that prevention and promotion are indispensable complements to care (2, 8). Crucially, a growing evidence base confirms that targeted positive psychology interventions can effectively enhance well-being, prevent distress and build resilience across the life course (10–15).

In sub-Saharan Africa, mental health resources remain scarce. A systematic review of nearly 98,000 young people reported median prevalences of 26.9% for depression and 29.8% for anxiety (16). Among South African employees, national surveys reveal that over half have a diagnosed mental health condition, one-third screen positive for depression and 30% experience significant psychological distress (17). Three-quarters struggle to “switch off” from work, and fewer than half who are offered Employee Assistance Programs ever use them (18). These data underscore the urgent need for upstream, context-sensitive interventions that can prevent distress before it reaches clinical levels.

Workplace interventions offer a strategic setting for prevention. Human Resource Management (HRM) is uniquely positioned to embed co-designed, multi-component programs that reshape harmful psychosocial environments and equip managers and peers to support well-being (19, 20). Structured interventions encompassing mindfulness, optimism, gratitude and resilience training have demonstrated both psychological and organizational benefits (10, 11, 13, 145) and can be delivered cost-effectively by non-clinical facilitators (21).

This study introduces *The Good Life* (TGL) training program, a culturally adapted intervention designed to cultivate employee well-being and engagement through a combination of positive psychology practices, structured peer interaction, and reflective exercises. Conceptually anchored in Seligman’s PERMA model (2011) and Fredrickson’s broaden-and-build theory (2001), TGL seeks to empower employees to proactively manage stress, build psychological resources, and foster social connections within the organizational context.

The Good Life (TGL) training program thus puts principle into practice within the organizational setting. By embedding a co-designed, multi-component intervention, TGL seeks not merely to treat stress or disengagement after they occur, but to reshape the very social and environmental factors that give rise to them. Through structured peer interaction, reflective exercises and skills for autonomy and connection, TGL embodies the Lancet’s call to move beyond treatment and toward a proactive, preventive model of mental-health stewardship in the workplace.

## 2 A brief literature review

### 2.1 The impact of poor mental health and well-being at the workplace

Poor employee well-being exacts a heavy toll on organizations, manifesting in reduced productivity, increased absenteeism and higher turnover (22–24). Meta-analytic evidence reveals a robust positive correlation between well-being and job performance: employees reporting higher life satisfaction and positive affect consistently deliver better quality work and customer service (25–28). Conversely, chronic stress and disengagement undermine cognitive function and motivation, eroding both individual and team outcomes (29, 30). Gallup’s (31) global survey identifies low engagement and high stress as two of the greatest organizational challenges worldwide, with highly engaged teams outperforming their counterparts by up to fivefold on key business metrics (29, 32). Moreover, the reciprocal relationship between engagement and well-being means that interventions targeting one domain often benefit the other (33, 34).

### 2.2 Why do we need an HR well-being program at work

Workplaces have become a critical battleground in the global struggle against rising mental-health challenges: the World Health Organization estimates that one in eight people suffers from a mental disorder, and depression alone now accounts for over 12 billion lost workdays and US $1 trillion in forgone productivity each year (7, 8, 35). Yet despite clear evidence that psychological well-being is even more predictive of job performance than physical health (36), HRM has historically focused on operational metrics and “hard” outcomes, leaving employee flourishing to chance or *ad hoc* wellness perks (19, 20). The result is striking: global employee engagement stagnated, and overall employee wellbeing declined, collectively costing economies some US$8.9 trillion annually, or 9 percent of global GDP (31). In South Africa, where this study’s intervention was tested, engagement and thriving rates are among the world’s lowest, and workplace stress contributes heavily to both absenteeism and turnover (31). Without a structured, well-being program embedded into core talent and leadership practices, organizations will continue to shoulder unsustainable human and economic costs. A targeted training intervention—co-developed with employees to reflect local cultural and organizational realities—is therefore essential not only to safeguard mental health but to unlock higher engagement, resilience, and long-term performance.

### 2.3 Conceptual foundation of well-being

Well-being is a multifaceted concept with no universally accepted definition, varying across disciplines and theoretical perspectives (37, 38). There is currently no unified theoretical framework explaining how employee well-being influences work performance, and there is a lack of consensus on the fundamental definitions of both well-being and performance (39). Reviews have categorized well-being into multiple dimensions: hedonic well-being, life satisfaction, eudaimonic well-being, mental well-being, social well-being, and physical health (40–42). WHO (7) recognizes mental health as an integral part of well-being and a fundamental human right. However, the lack of a universal definition contributes to inconsistencies in conceptualization and measurement, making it essential to adopt a structured approach to studying workplace well-being (43).

The measurement of well-being is equally diverse, with at least 69 well-being scales identified across different disciplines (42). Another scoping review identified 109 workplace mental health instruments (44). Given this variability, this study focuses on three widely used dimensions of workplace well-being: life satisfaction, flourishing, and mental well-being. These are measured using the Satisfaction With Life Scale (SWLS), the Flourishing Scale, and the Warwick-Edinburgh Mental Well-being Scale (WEMWBS), respectively (40–42, 45). Together, these constructs evaluate well-being, integrating cognitive assessments of life, psychological resilience and broader personal development.

Beyond well-being, this study also considers work engagement and stress, which significantly impact workplace productivity and employee functioning. Engagement, measured using the Utrecht Work Engagement Scale (UWES), is consistently linked to higher job performance and reduced turnover (31). Similarly, stress, assessed through the Perceived Stress Scale (PSS), plays a crucial role in burnout, workplace efficiency, and mental health outcomes (46, 47). By integrating these measures, this study ensures a holistic assessment of workplace well-being, allowing for a nuanced understanding of how well-being interventions impact employees’ engagement, stress levels, and overall workplace experience.

### 2.4 Designing a HR well-being training program

**Figure 1:**
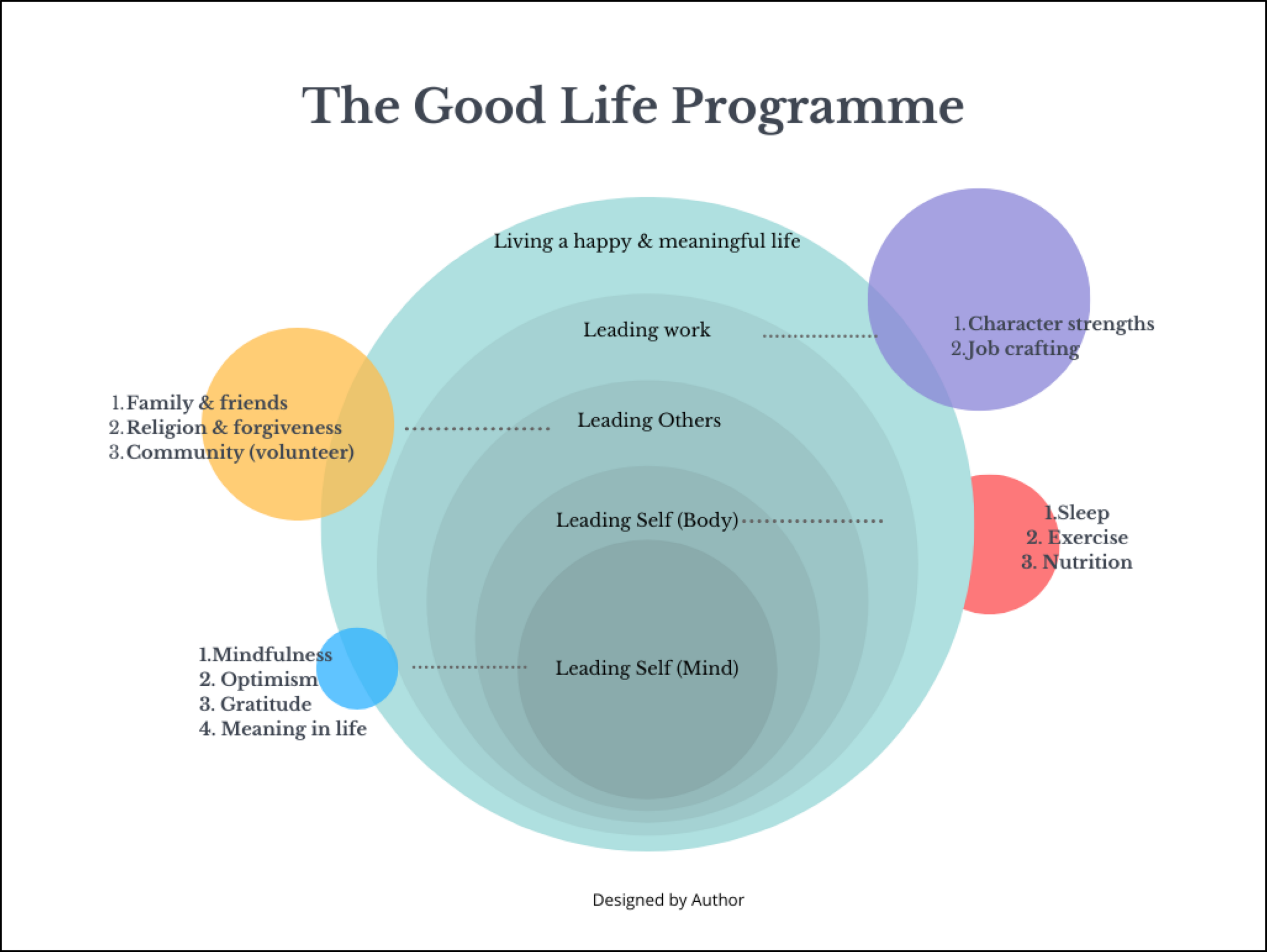
HR training program: a concentric framework of self-leadership, social connection, and workplace well-being (authors’ own compilation).

*The Good Life* training program was developed as a structured, multi-component HR intervention aimed at enhancing workplace well-being, employee engagement, and stress management (see Figure 1). The design process was participatory and grounded in both theory and employee input. This participatory process unfolded over three phases: evidence-based construct selection via literature review, pilot testing with a diverse set of employees, and a focus group discussion to co-refine the content. For instance, ‘relationships’ was added and ‘forgiveness’ was retained based on strong participant endorsement during the focus group. This ensured the intervention aligned with both scientific evidence and workplace realities. The authors (who are also HR professionals) developed detailed operational guidance, learning activities, and evidence-based tools to ensure real-world applicability. The session structure and key theoretical anchors are summarized in Table 1. Unlike many theoretical models, *The Good Life* offers a practitioner-ready, scalable intervention that addresses the gap between psychological theory and Human Resource Development (HRD) practice, especially in low- and middle-income settings.

**Table 1:** Session structure and key theoretical anchors for the well-being program (authors’ own compilation).

Session	Theme	Content	Theoretical anchors
1	Introduction	Introduction to team members. Well-being, facts and myths about happiness. Why we are not happy?	Myths about happiness – (95). Well-being formula and focus on Voluntary (V) activities (52).
2	Leading Self	Mindfulness and savoring. Introduction to mobile apps, practices and various breathwork session on YouTube.	Mindfulness – (96–99). Savoring – (100–106).
3	Leading Self	Gratitude: Reflect and write down three things that you are thankful for in life. Write a thank-you note for one person in your life. Introduction to free gratitude mobile apps.	Reflections on gratitude – (107–112)
4	Leading Self	Forgiveness and Optimism: Write one forgiveness letter for self or other. Ways to enhance positive outlook. Introduction to learned helplessness.	Forgiveness – (113–118). Optimism – (119–122)
5	Leading Self	Physical activity, sleep and nutrition. Various Ted talk videos on these subjects.	Physical activity – (123). Sleep – (124, 125). Nutrition – (126, 127).
6	Leading Others	Relationship and being pro-social: Developing strategy to build close relationships. Case-Harvard Adult Development study and findings/Ted Talk.	Building strong relationships – (128–134)
7	Leading at Work	Signature Strength and Job Crafting exercises.	Strength – (135, 136) Job crafting – (137–139, 142)
8	Conclusion	Meaning in life: Developing an integrated plan for a healthy and meaningful life. Using workbook to create a personal plan.	Finding purpose in life – (140, 141)

### 2.5 Theoretical framework

**Figure 2:**
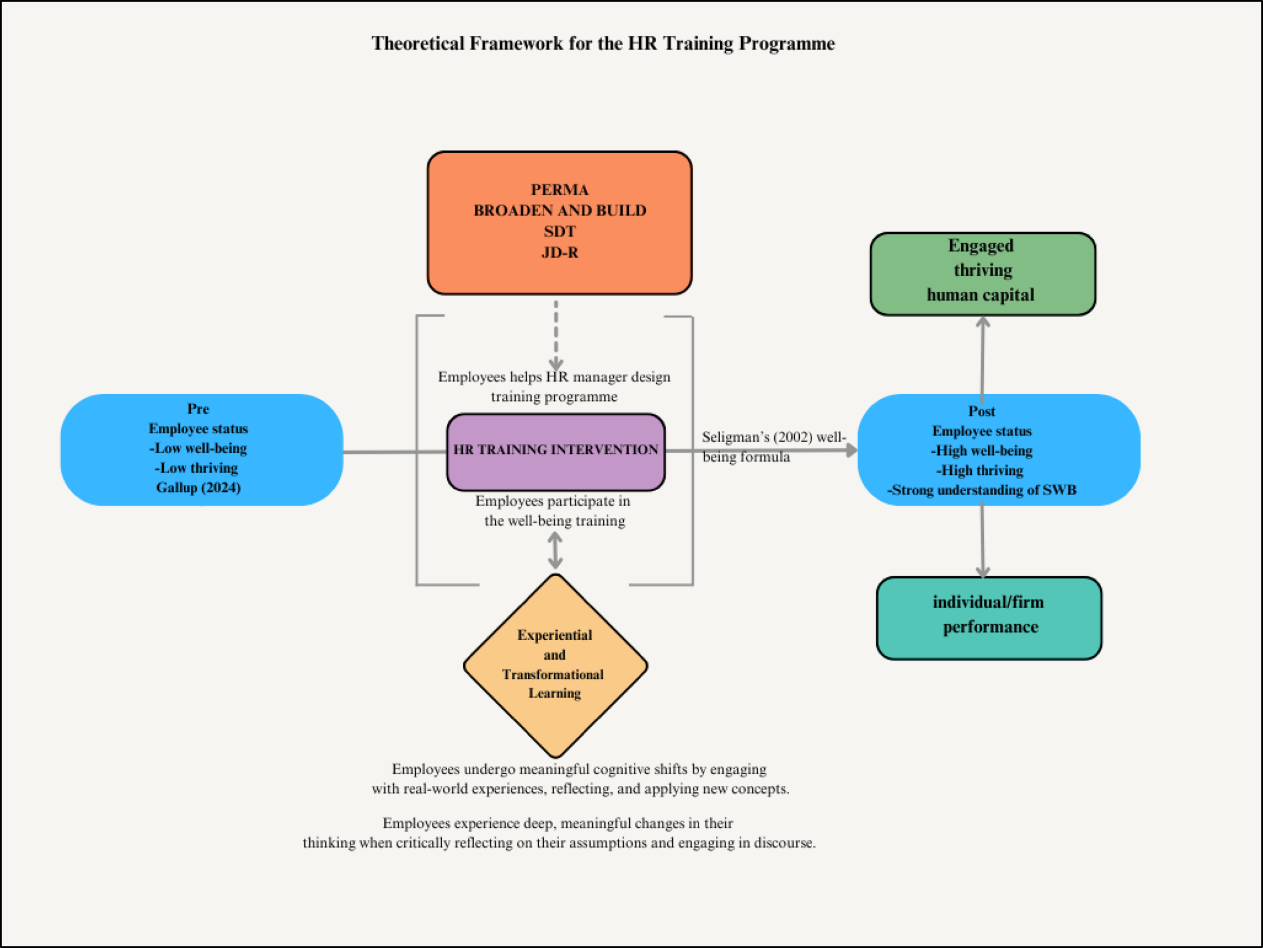
Theoretical framework for the well-being within HRM and HRD (authors’ own construction).

The theoretical framework presented in Figure 2 integrates key concepts from well-being science, HRM, and HRD to guide the design and delivery of the training intervention. It positions well-being as a multidimensional construct, developed through structured learning and embedded in organizational practice to enhance both individual thriving and firm-level performance.

At the center of the framework is the HR training program designed to address three key challenges facing modern workplaces: low engagement, high stress, and poor well-being. Grounded in both experiential learning and transformational learning theories, the intervention aims to create opportunities for employees to reflect on their lived experiences, question limiting beliefs, and adopt new, evidence-based strategies for improving well-being. This learning is not abstract but applied through real-world exercises and peer interactions that encourage behavioral change and deep personal insight.

The training intervention draws from multiple well-established psychological frameworks to support its design and outcomes. These include PERMA (48), the broaden-and-build theory (49), self-determination theory (SDT) (50), and the job demands–resources (JD-R) model (51). Together, these theories underscore the importance of positive emotions, intrinsic motivation, autonomy, and personal resources in driving engagement and resilience in the workplace.

Seligman’s (52) well-being formula—H = S + C + V— informs the HR program’s focus on voluntary activities (V), which represent an area where individuals can intentionally foster greater well-being. While set-point (S) and life circumstances (C) are relatively fixed, voluntary actions such as gratitude, mindfulness, social connection, and strength-based reflection can be shaped through targeted learning experiences. These activities are integrated into the HR training curriculum through experiential modules that allow employees to practice new behaviors, reflect on outcomes, and iterate on their strategies.

These voluntary activities are not isolated practices but are woven into the program’s learning architecture, fostering deeper change through transformational learning (53). Employees are encouraged to critically examine their assumptions about stress, work, and personal capabilities, shifting from low awareness to a high understanding of well-being and its applications at work. In parallel, experiential learning (54) is applied through real-time learning cycles that involve concrete experience, reflective observation, abstract conceptualization, and active experimentation.

Further, the program introduces employees to multiple constructs like building relationships, signature strengths and job crafting—that directly support the JD-R and SDT framework by enhancing personal resources. Teaching employees how to identify and apply their core strengths increases competence and fulfillment, while job crafting enables them to proactively reshape their roles to better align with their values and skills. These interventions not only mitigate the negative effects of job demands but also foster motivation, engagement, and psychological resilience.

## 3 Methodology

This study evaluated the effectiveness of a training intervention using an exploratory, quasi-experimental, explanatory sequential mixed-methods design, where quantitative data were collected first over 3 months, followed by qualitative insights (generated by interviewing a small cohort of diverse participants at the end of the study) to provide a deeper understanding of the findings. This phase measured improvements in well-being, work engagement, and stress while comparing the effectiveness of classroom-based and online training formats.

Verhoef and Casebeer (55) highlight that most health studies primarily rely on quantitative methods to assess whether significant improvements in well-being have occurred. However, these studies often fail to explain an intervention’s success or failure. Therefore, a brief exploratory qualitative phase was incorporated after the intervention to further explore participants’ experiences and contextual factors influencing program outcomes. This approach ensures a more comprehensive evaluation by integrating quantitative outcome data with qualitative insights, making Mixed Methods the most suitable choice for assessing the well-being program (56). This approach aligns with previous studies that employed similar exploratory mixed-methods designs to evaluate workplace interventions, integrating quantitative assessments with qualitative insights to capture both outcomes and participant experiences (57–60).

While the study employed a quasi-experimental design without a control group, statistical methods such as within-group analyses and effect size calculations were utilized to assess intervention impact. The exploratory nature of the study also justifies the absence of a control group, as the primary objective was to identify emerging patterns and mechanisms rather than to establish definitive causal inferences. Future studies should incorporate a randomized control group to strengthen causal inferences and mitigate potential confounding effects.

### 3.1 Research philosophy and approach

The study adopts a pragmatic research philosophy (61, 62), recognizing that workplace well-being is shaped by multiple, context-dependent realities (ontology), and that knowledge emerges through an iterative blend of quantitative surveys and qualitative interviews (epistemology). It also embraces researcher values and ethical considerations as integral to the inquiry (axiology). By first measuring well-being outcomes statistically and then exploring participants’ experiences qualitatively, the explanatory sequential mixed-methods design ensures the training program is both evidence-based and responsive to employees’ lived contexts.

### 3.2 Research participants and sampling

The study was conducted in a South African pharmaceutical firm employing 108 individuals, predominantly female and largely based in the sales department (Table 2). From this workforce, 95 employees (excluding HR and senior management) were invited to participate via non-probability convenience sampling. Of these, 87 gave informed consent and completed the pre-intervention survey. Participants were allocated into three distinct intervention cohorts: two classroom-based groups (one delivered over two full days and the other over four half-days across 4 weeks), and an online group delivered through four half-day sessions spread over 4 weeks, each comprising 16 contact hours in total. Initially, a control group of 15 participants was planned and kept on a waitlist for 6 months; however, this approach was subsequently discarded as control participants frequently interacted with the experimental groups, potentially biasing results. Moreover, significant attrition occurred within this control group, as approximately eight out of the original 15 participants left the company during the intervention year. Of the original 69 participants who attended the training, only 50 completed the follow-up survey 3 months post-intervention. Those participants who did not complete the follow-up survey were excluded from the analysis.

**Table 2:** An overview of the training groups (authors’ own construction).

Details	Group 1	Group 2	Group 3	Total
Group type	Classroom	Classroom	Online	
Format	2 full days	4 half days	4 half days	
Participants invited	95			95
Participants who gave consent	87			87
Survey completed before training	25	20	24	69
Survey completed 3 months + after training	19	14	17	50

The use of non-probability sampling was appropriate given the study’s focus on a specific organizational setting where logistical and operational constraints necessitated targeted recruitment. This approach facilitated access to a representative cross-section of employees across various roles and work locations, aligning with the study’s objective to evaluate differential responses to training delivery formats (i.e., in-person versus online). While this approach facilitated targeted recruitment within a specific workplace context, it may limit the generalisability of findings to other organizational settings. Future studies should consider stratified or random sampling to enhance representativeness.

To gain a deeper understanding of participants’ experiences with the training intervention, semi-structured interviews were conducted with a purposive subsample of intervention participants 3–6 months after completion. A total of 15 employees (five from each training cohort) were selected to ensure representation across delivery modes (two-day intensive classroom, four half-day classroom, and four half-day online) and roles (sales versus non-sales). Interviewees were purposively chosen based on demographic variation (gender, tenure, and job grade) and quantitative outcomes (high versus low change in WEMWBS scores) to capture diverse perspectives regarding program impacts and barriers to behavioral change. This sample size aligns with recent findings by Squire et al. (63), who demonstrated that a sample of 15–23 participants typically achieves saturation in qualitative interviews, indicating minimal benefit in collecting additional data beyond this point.

### 3.3 Data collection

The training program evaluation employed an explanatory sequential mixed-methods design grounded in pragmatism (61), whereby quantitative pre- and post-intervention surveys first established the statistical effects of the program on well-being, engagement and stress; this was subsequently followed 6 months later by semi-structured interviews to elucidate participants’ lived experiences and to understand why certain employees exhibited stronger or weaker responses; finally, these quantitative and qualitative findings were integrated to formulate practical, HR-driven recommendations for optimizing future workplace well-being interventions.

Quantitative data collection for the training program evaluation relied on five validated instruments—the Perceived Stress Scale (PSS-4), the Utrecht Work Engagement Scale (UWES-3), the Satisfaction With Life Scale (SWLS), the Flourishing Scale (FS) and the Warwick–Edinburgh Mental Well-Being Scale (WEMWBS-14)—administered online at two key time points: immediately before the intervention and 3 months afterwards. Each survey incorporated informed-consent, confidentiality and opt-out provisions to ensure ethical compliance, yielding comprehensive data on respondents’ perceived stress, work engagement and overall well-being.

Specifically, each instrument was selected on the basis of its robust psychometric properties and relevance to workplace well-being. The PSS is among the most widely used measures of perceived stress, capturing respondents’ appraisal of unpredictability, uncontrollability and overload in daily life (64–67). Work engagement was assessed with the ultra-short UWES-3, which operationalizes engagement as a positive, fulfilling work-related state reflected in vigor, dedication and absorption; this version demonstrates excellent reliability and valid associations with job resources and satisfaction, while showing minimal correlation with burnout (68–70). Life satisfaction was measured by the SWLS, a concise instrument favored for non-clinical populations that correlates with quality-of-life dimensions such as anxiety, optimism and sleep quality (71, 72). The FS provided a single eudaimonic well-being score based on eight items addressing relationships, optimism, self-esteem and purpose (73–75). Finally, the WEMWBS-14 comprises 14 positively phrased items scored on a five-point Likert scale (14–70), with higher scores indicating better mental health; which exhibits strong convergent validity, no ceiling effects and cultural applicability in the South African sample (76–79). The specific variables, their operationalization and classification are summarized in Table 3.

**Table 3:** Various constructs and operationalization (author’s own construction).

Variable	Operationalization	Type
Training Intervention	Training program to enhance well-being, work engagement and stress resilience	Independent variable
Stress	Perceived Stress Scale (PSS-4)	Dependent variable
Engagement at work	Utrecht Work Engagement Scale (UWES-3)	Dependent variable
Well-being	Warwick-Edinburgh Mental Well-being scale (WEMWBS) Satisfaction with Life Scale (SWLS) Flourishing Scale	Dependent variable

Qualitative data were also gathered via semi-structured interviews with a purposive subsample of 15 participants. Each 45–60 min interview followed a standardized guide, explored participants’ motivations for enrolling, perceived benefits and challenges, and suggestions for improving the program, and was audio-recorded with informed consent.

### 3.4 Data trustworthiness

Data trustworthiness was established through multiple strategies to ensure credibility, dependability, confirmability, and transferability in the mixed-methods design. Credibility was enhanced by triangulating quantitative findings with qualitative interview data, allowing for cross-validation of well-being outcomes through participants’ reflections on the intervention. Verbatim quotations further substantiated key themes, providing direct evidence of participants’ lived experiences. Dependability was reinforced by maintaining a comprehensive audit trail that documented each phase of data collection and analysis, with independent coding checks conducted to verify theme consistency. Confirmability was addressed through reflexive memos recorded throughout the qualitative analysis to mitigate researcher bias and ensure neutrality. Additionally, the coding framework was independently reviewed by the co-authors, who provided feedback on theme coherence and consistency. Transferability was supported by providing rich, thick descriptions of the research context, participant characteristics, and intervention delivery, enabling readers to assess the applicability of findings to similar workplace settings. These measures collectively contributed to a systematic and transparent qualitative process, reinforcing the credibility and reliability of the findings.

### 3.5 Data analysis

Quantitative analyses were conducted in SPSS using a three-step non-parametric approach. First, Shapiro–Wilk tests (*α* = 0.05) assessed normality for each outcome and time point, confirming violations of normality assumptions for key well-being indicators [(80, 81); see Supplementary Table S1]. Second, within-group changes from pre- to three-month follow-up were evaluated with Wilcoxon signed-rank tests, and between-group differences across delivery formats at each time point were examined with Mann–Whitney *U* tests. Third, for the subset of participants with three measurements (pre-training, immediate post-training, three-month follow-up), Friedman tests assessed overall time effects, with post-hoc Wilcoxon signed-rank comparisons pinpointing significant pairwise contrasts (see Supplementary Tables S3–S5). All tests used a two-tailed *α* < 0.05 and reported exact *p*-values without Bonferroni correction to preserve statistical power in these small samples (82). Rank-biserial correlations (*r*) quantified effect sizes for all Wilcoxon and Mann–Whitney contrasts. Descriptive statistics—including medians, interquartile ranges, means, standard deviations, skewness, kurtosis and frequencies—summarized outcomes and demographics (see Supplementary Table S2), and Cronbach’s *α* coefficients established internal consistency for each scale. Cases with missing data were omitted via a complete-case approach, and data integrity was ensured by cross-checking SPSS imports against the original Excel files.

Qualitative interview transcripts were analyzed manually using thematic analysis (83). After familiarization through repeated readings, the researcher applied open coding (e.g., “increased motivation,” “practical application,” “emotional control”) to salient passages. Codes were then clustered into higher-order themes (e.g., “enhanced self-awareness and emotional resilience”), iteratively reviewed for coherence, and supported with verbatim quotations. Themes were reviewed against the raw data in multiple iterations, with detailed reflexive memos maintained throughout to ensure coherence and credibility.

Quantitative and qualitative data were synthesized during the final phase of analysis, enabling the integration of statistical outcomes with participant narratives to contextualize the observed intervention effects. This approach facilitated the identification of mechanisms underlying observed changes in well-being and engagement, contributing to a more nuanced interpretation of program effectiveness.

Quantitative and qualitative data were synthesized through narrative synthesis, whereby thematic findings from the qualitative phase were juxtaposed against statistical outcomes to identify areas of convergence and divergence. This integrative approach enabled a comprehensive interpretation of how the TGL training program influenced well-being and engagement, while also highlighting potential mechanisms of change underlying observed outcomes.

### 3.6 Ethical considerations

Key ethical safeguards were embedded throughout the quantitative phase to protect participants’ rights and well-being. Participation was entirely voluntary, with a clear right to withdraw at any time without repercussion, as specified in the online consent form. Before completing each survey, employees received comprehensive information on the study’s aims, duration, potential risks and anticipated benefits. To ensure anonymity and data security, responses were collected anonymously with support from the University of Johannesburg’s Statistics department and stored on a password-protected server accessible only to the main researcher and authorized co-authors. Measures were also taken to minimize survey fatigue and avoid psychological harm by spacing follow-up assessments and allowing participants to skip questions without penalty. Finally, although the pharmaceutical company’s HR department facilitated recruitment, care was taken to avoid any perception of coercion, ensuring that no employee felt obliged to participate.

In the qualitative phase, additional ethical safeguards were implemented to protect participant confidentiality and manage sensitive disclosures. Participants were informed of their right to withdraw from the interview at any stage without penalty, and all notes were securely stored on a password-protected server accessible only to the researcher.

## 4 Findings

This section summarizes the impact of three delivery formats of The Good Life program in South Africa—online, two full-day classroom, and four half-day classroom—on employee well-being (WEMWBS), engagement (UWES-3) and secondary outcomes (SWLS, FS, PSS-4) over 3 months. Non-parametric tests were used throughout (see Methods).

### 4.1 Well-being (WEMWBS)

- Two full-day classroom (*n* = 19): Median WEMWBS rose by 7.0 points, *Z* = −2.474, *p* = 0.013, *r* = 0.57 (strong effect).
- Online (*n* = 17): Median increase of 1.0 point, *Z* = −2.190, *p* = 0.029, *r* = 0.53 (strong effect).
- Four half-day classroom (*n* = 14): No significant change, *p* = 0.755.

### 4.2 Engagement (UWES-3)

- Two full-day classroom: Median UWES gain of 0.8 points, *Z* = −2.378, *p* = 0.017, *r* = 0.55 (strong effect).
- Four half-day classroom: Moderate increase, *Z* = −1.699, *p* = 0.089, *r* = 0.44 (moderate trend).
- Online: No significant change, *p* = 0.585.

### 4.3 Secondary outcomes (SWLS, FS, PSS-4)

- Two full-day classroom: Positive trends in life satisfaction (SWLS, *p* = 0.055, *r* = 0.44), flourishing (FS, *p* = 0.075, *r* = 0.41) and stress reduction (PSS-4, *p* = 0.053, *r* = 0.44)—all moderate effects.
- Online and four half-day formats: No significant or moderate trends (all *p* > 0.10).

Overall, the two full-day classroom format emerged as the most effective delivery method, demonstrating moderate to strong effects across all primary and secondary outcomes. Specifically, significant improvements were observed in well-being (*r* = 0.57), engagement (*r* = 0.55), and stress reduction (*r* = 0.44), with effect sizes indicating practical significance. Conversely, the online format produced a modest gain in well-being (*r* = 0.53), while the fragmented half-day format yielded no significant changes across any outcomes, suggesting that continuity and immersive learning are critical to intervention success.

### 4.4 Summary of findings

- Strong Significant Changes (*p* ≤ 0.05):
- Online: WEMWBS
- Two full-day classroom: WEMWBS, UWES-3
- Moderate Changes (0.05 < *p* ≤ 0.10):
- Two full-day classroom: SWLS, FS, PSS-4
- Four half-day classroom: UWES-3

Overall, intensive in-person training (two full days) yielded the most consistent and largest improvements across well-being, engagement and related outcomes. The online format improved well-being only, while the half-day classroom format produced a moderate boost in engagement but no reliable changes in well-being or secondary measures.

In interpreting effect sizes, small effects (*r* ≈ 0.10–0.30) suggest minimal practical impact, moderate effects (*r* ≈ 0.31–0.50) indicate noticeable but limited changes, and strong effects (*r* > 0.50) reflect substantial and potentially impactful outcomes (143). In this study, the two full-day format produced strong effect sizes for well-being (*r* = 0.57) and engagement (*r* = 0.55), underscoring its effectiveness relative to other formats.

Qualitative themes of immersive peer interaction, structured reflection and managerial support help explain why the full-day format was most effective.

#### 4.4.1 Additional data provided in the supplement

Supplementary Table S1 presents the Shapiro–Wilk test statistics, sample sizes and *p*-values for each outcome measure at pre-training (T₁) and three-month follow-up (T₃) across delivery formats, indicating which distributions significantly departed from normality.

Supplementary Table S2 presents the median and interquartile range for each well-being and engagement measure at baseline and three-month follow-up, by training format.

Supplementary Table S3 presents the Kruskal–Wallis *H* statistics, degrees of freedom and *p*-values for between-group comparisons of each outcome measure at pre-training (T₁) and three-month follow-up (T₃), indicating which differences across delivery formats reached statistical significance.

Supplementary Table S4 presents Friedman *χ*^2^ statistics, degrees of freedom and *p*-values for within-group comparisons of each outcome measure across three time points, indicating which changes over time reached statistical significance.

Supplementary Table S5 presents Wilcoxon *Z* statistics, sample sizes (*N*) and rank-biserial correlations (*r*) for pre- to three-month follow-up comparisons (T₁ to T₃) for each outcome measure across delivery formats, illustrating the magnitude of within-group changes.

In addition to tabular summaries, Supplementary Figure S1 presents boxplots of each outcome measure at T₁ and T₃ by training format, offering a visual comparison of score distributions and highlighting changes over time.

#### 4.4.2 Integrating quantitative and qualitative findings: understanding what worked and why

To explain *why* certain delivery formats succeeded, we merged key non-parametric outcomes with thematic interview data in Table 4. This joint display focuses exclusively on the three South African cohorts and ensures consistency with our supplementary statistics.

**Table 4:** Mixing of qualitative with quantitative findings (authors’ own construction).

Group type	Significant statistical change	Qualitative insights (reasons why or why not)
SA Classroom (2 Full Days)	WEMWBS (*p* = 0.013), UWES (*p* = 0.017)	High engagement due to immersive sessions, uninterrupted learning time, and active discussions. Some participants appreciated employer support for giving time off for 2 full days. *“This training reminded me how important it is to care for myself. I liked the analogy of being in a plane, and when face masks drop, we need to wear them first and thereafter assist our loved ones. We cannot make others happy if we are not happy. We have to prioritize ourselves.”* Participant, Supply chain head, Male. *“Hearing others’ experiences helped me realize I’m not the only one battling these demons. It was comforting and supportive.”* Participant, Quality, Male. *“Mindfulness and being positive in life are good skills to learn. It was good to know that these things can be learnt.”* Participant, Sales, Male.
SA Online (4 Half Days)	WEMWBS (*p* = 0.029)	Participants who engaged actively saw improvements. However, some struggled with distractions. Managers requiring camera-on policy enhanced engagement with content. *“The program taught me to control my stress better. I’m less reactive now and can handle difficult situations more calmly.”* Participant, Sales, Female. *“I never realized how much impact small changes could make. I’m now more motivated to take daily actions for my happiness.”* Participant, Marketing, Female. *“It was nice understanding the science of well-being. My focus has shifted on family and friends.”* Participant, Female, Sales
SA Classroom (4 Half Days)	No significant change	Participants struggled with workload balance, leading to resentment. Afternoon sessions disrupted routines, making focus difficult. *“It is tough to attend the program after work. We are too drained to fully commit to this workshop after a full day of sales meetings”* Participant, Sales representative, Male. *“It is important for management to make the program more accessible regarding time slots. It is unfair for sales managers to make us attend any workshop after the day-long work.”* Participant, Sales representative, Female. *“The content was good but condensing it a bit would make it easier to absorb.”* (Participant, Sales representative, Female).

To contextualize the observed statistical outcomes, qualitative insights were integrated to elucidate why certain delivery formats were more effective than others. Participants in the two full-day classroom format reported feeling more connected to peers and more engaged with the content, attributing improvements in well-being to structured reflection and uninterrupted learning time. In contrast, those in the four half-day classroom sessions expressed frustration with scheduling conflicts and mental fatigue, factors that may have undermined intervention efficacy despite content quality. These insights provide a nuanced understanding of how delivery format influences both engagement and perceived well-being benefits.

This mixed-methods integration demonstrates that intensive, in-person delivery produced the largest and most consistent improvements, driven by immersive peer learning and explicit managerial support, while the online format achieved modest well-being gains when structured engagement strategies were enforced. The fragmented half-day classroom format failed to create the focused learning environment necessary for meaningful change.

Although some peer learning occurred, and a few participants enjoyed the sessions, overall participation was low, leading to minimal individual improvements in WEMWBS scores. The findings suggest that for well-being training to be effective, employees must be fully present and engaged, without the added strain of completing work tasks beforehand.

## 5 Discussion

This study set out to evaluate whether a training program (TGL) produced genuine improvements in employee well-being and engagement, rather than merely reflecting pre-existing associations. By combining a longitudinal, quasi-experimental design with in-depth interviews, we can move beyond correlation to draw cautious inferences about causality.

Three meta-analyses provide context for interpreting the well-being effect sizes observed in this study. Sin and Lyubomirsky (84) reported a moderate effect size of *r* = 0.29 for well-being in a broad set of positive psychology interventions, while Bolier et al. (85) found a smaller effect size of *d* = 0.34 in a more rigorously controlled set of randomized controlled trials. More recently, Hendriks et al. (86) reported effect sizes ranging from *g* = 0.24–0.34 for well-being in multi-component interventions, noting higher effects in non-Western settings. In comparison, the effect size observed in the two-day classroom format of the TGL intervention (*r* = 0.55) substantially exceeds these averages, suggesting that intensive, immersive training formats may be particularly effective in workplace well-being interventions.

The quantitative findings indicating that the two-day classroom format yielded the largest gains in well-being (WEMWBS) and engagement (UWES) are further elucidated by qualitative narratives that underscore the unique advantages of this immersive delivery. Participants consistently highlighted the uninterrupted nature of the training, which allowed them to fully engage with well-being practices like mindfulness and gratitude journaling without workplace distractions. Unlike the fragmented (half-day) and online formats, the two-day structure fostered a cohesive learning environment where participants could openly share personal experiences and challenges, facilitating deeper connections and peer learning. Managerial support also played a critical role; employees perceived the provision of two full days off for training as a strong organizational commitment to their well-being, enhancing their sense of value and motivation to apply the learned strategies. Furthermore, the classroom setting enabled immediate feedback and richer discussions, reinforcing key well-being concepts through real-life examples. In contrast, the half-day format disrupted these cycles, with participants expressing frustration over balancing work demands with training, leading to lower engagement and less effective integration of well-being practices.

Nonetheless, several threats to causal inference warrant acknowledgement. The absence of a contemporaneous, fully isolated control group means we cannot entirely rule out organizational or seasonal influences on well-being. Non-random participant allocation—driven by operational constraints and managerial discretion—may have introduced selection bias, as more motivated or available employees disproportionately entered the intensive formats. Finally, repeated measurement raises the possibility of testing effects, whereby mere exposure to well-being questions enhances self-awareness independently of the training.

Balancing these considerations, we conclude that there is strong, mixed-methods evidence to support a cautious causal claim: the TGL program, when delivered as a sustained, immersive workshop, meaningfully enhances employee well-being and engagement. However, this finding must be interpreted in light of the quasi-experimental constraints. Future research employing randomized assignment, active control conditions and strategies to minimize attrition will be essential to confirm and extend these results.

### 5.1 Theoretical integration: why immersive formats worked

Our findings align closely with the theoretical framework (Figure 2), which integrates PERMA, broaden-and-build, SDT, and JD-R models underpinned by experiential and transformational learning. The two-day, in-person workshops—the format that produced the largest WEMWBS and UWES gains—enabled uninterrupted cycles of concrete experience, reflective observation, abstract conceptualization, and active experimentation (54). This format allowed participants to fully engage in well-being practices, fostering positive emotions (PERMA) through structured activities such as gratitude journaling, strengths identification, and meaning-focused reflections. These activities not only broadened cognitive and social resources (49) but also addressed core SDT needs for autonomy, competence, and relatedness (50).

Moreover, the integration of PERMA constructs (gratitude, character strengths, relationships, meaning) effectively functioned as job resources within the JD-R model (51), equipping employees with personal and social resources to buffer job demands and sustain well-being. The full-day format further facilitated job crafting and peer support, reinforcing the application of well-being strategies in the workplace.

In contrast, the half-day and online formats fragmented these learning cycles, limiting opportunities to internalize well-being practices, as similarly found by Stratton et al. (94). Participants in these formats reported struggling to maintain focus amid work-related distractions, reducing the impact of voluntary activities (e.g., mindfulness, gratitude) that Seligman (52) identifies as drivers of sustainable happiness. This disparity underscores the importance of immersive, uninterrupted training in embedding well-being practices, particularly in high-demand workplace settings.

Thus, the mixed-methods data suggest that dedicated, intensive training time—supported by organizational endorsement—enhances the translation of theoretical frameworks into tangible workplace well-being outcomes, highlighting the critical role of experiential and transformational learning in HRD.

### 5.2 Positioning the training program’s effectiveness within existing research

Meta-analyses and systematic reviews consistently demonstrate that well-being interventions yield moderate to large improvements in mental health, life satisfaction and psychological functioning in both clinical and occupational settings (86–89). Meanwhile, the TGL training program extends this evidence by showing that delivery format, organizational context and participant engagement critically shape outcomes. In particular, our finding that full-day classroom delivery produced larger improvements in WEMWBS and UWES scores than the online and half-day formats underlines the importance of immersive, interactive approaches.

### 5.3 Addressing geographic and methodological gaps

Previous reviews have highlighted a pronounced WEIRD (Western, Educated, Industrialized, Rich, and Democratic) bias in positive psychology interventions and a paucity of workplace studies in African settings (90–92, 144). By implementing and evaluating a structured, program in South Africa, this study responds directly to those calls. Moreover, whereas many workplace interventions rely solely on randomized trials, TGL adopts an explanatory sequential mixed-methods design to integrate quantitative pre–post measures with qualitative interviews, thereby offering richer causal insights (47, 89).

## 6 Practical implications

For HR practitioners in low- and middle-income contexts, TGL offers a ready-to-implement framework that bridges psychological theory and everyday HRD practice. The strong effect sizes observed for full-day delivery underscore the value of investing in dedicated training time, even amid resource constraints. Organizations should consider hybrid models that combine face-to-face immersion with digital supports to balance cost and impact.

However, the effectiveness of such interventions depends not only on content and delivery but also on the broader organizational environment in which they are applied. As highlighted by the JD-R model (51), personal resources gained through training may have limited impact if employees continue to face high job demands and lack the autonomy or support to implement new behaviors. Even when well-intentioned, employees may struggle to apply well-being strategies in the face of time pressure or unsupportive work structures. To enable lasting change, HR professionals must pair such programs with structural enablers—such as manageable workloads, protected time for practice, and ongoing managerial support—that help embed well-being into the flow of daily work.

## 7 Limitations and recommendations

Despite yielding important insights, this study has several limitations. First, the absence of a control group limits causal inference; while the mixed-methods design partially triangulated effects, a future randomized controlled trial would provide stronger evidence of program impact. Second, variability in participant engagement and facilitator style across delivery formats may have influenced outcomes. Although standardized materials (workbook and slide deck) were used, high-engagement groups (e.g., two full-day classroom) often deviated from the planned curriculum, whereas lower-participation cohorts (e.g., online group) adhered strictly, confounding the effect of format with group dynamics. Third, longitudinal survey fatigue led to multiple dropouts from survey filling. The extensive battery of validated scales likely contributed to non-completion; briefer or staggered assessments may improve retention in future studies. Finally, reliance on self-report instruments invites response bias (e.g., social desirability, mood effects); integrating objective measures—such as absenteeism records or physiological markers (e.g., heart-rate variability)—would yield a more robust evaluation of well-being and stress reduction.

While the TGL program demonstrated promising improvements in individual well-being, a key limitation lies in the risk of unintentionally placing the responsibility for well-being solely on employees. This concern is particularly salient in organizations where HR professionals may not have formal training in psychological theory or organizational development. Although the intervention was grounded in the JD-R framework (51) to build personal resources such as optimism and resilience, its long-term impact ultimately depends on the broader work environment. Research by Van Wingerden et al. (93) underscores that employees’ ability to benefit from resource-building interventions—like job crafting—is closely linked to their sense of autonomy and the supportiveness of the organizational context. Without a work environment that reinforces and sustains these efforts, the effects of such interventions may diminish over time. We therefore recommend that well-being programs be embedded within a broader strategy that includes supportive leadership, equitable workload distribution, and enabling job design to foster sustainable improvements.

## 8 Conclusion

This study presents quasi-experimental evidence that a structured, well-being intervention—The Good Life—can effectively enhance employee mental well-being and engagement in non-Western workplace settings. The two full-day, in-person workshops generated the most substantial improvements in WEMWBS and UWES scores, underscoring the value of immersive, uninterrupted training formats. In contrast, online delivery produced more modest gains, suggesting that interactional depth and sustained peer engagement are critical for well-being outcomes.

By integrating validated psychometric measures with thematic analysis, the study addresses key geographical and methodological gaps in workplace well-being research, demonstrating how programs can be adapted to diverse cultural and organizational contexts. For organizations, the findings highlight the importance of allocating dedicated training time and managerial support to maximize program impact. Implementing hybrid models that combine in-person sessions with digital follow-ups may further sustain engagement while optimizing resources.

Future research should incorporate randomized controls, larger sample sizes, and objective measures (e.g., absenteeism, heart-rate variability) to confirm the intervention’s long-term effects and mitigate potential response biases. The Good Life provides a scalable, replicable model for embedding structured well-being initiatives into HR practice, linking employee development with broader organizational outcomes.

While experiential and transformational learning theories help explain the success of the two-day immersive format, cultural learning preferences may also play a role. Particularly in the South African workplace context, collectivist values and relational engagement styles may make participants more receptive to extended, in-person group formats that allow for connection, reflection, and dialog. Future research could further explore how such cultural dimensions shape delivery preferences and learning effectiveness in well-being interventions.

Future research should also explore the broader organizational outcomes of such interventions, particularly their effect on absenteeism, presenteeism, and productivity. While our study focused on psychological and experiential outcomes, workplace interventions must ultimately demonstrate relevance to organizational performance. Additionally, the effects of digital mental health interventions on absenteeism and presenteeism are often limited or non-significant, even when small gains in engagement and productivity are observed. This highlights the need for longitudinal and multi-level evaluation designs that integrate psychological, behavioral, and performance-related indicators to fully assess the impact of workplace well-being programs.

## Data Availability

The raw data supporting the conclusions of this article will be made available by the authors, without undue reservation.
