# Supplementary material for: Enhancing employee well-being through a culturally adapted training program: a mixed-methods study in South Africa
Source: Front Public Health. 2025 Aug 18;13:1627464. doi: 10.3389/fpubh.2025.1627464 (PMC12399526; doi:10.3389/fpubh.2025.1627464)
Supplement: Supplementary file 1 [file Supplementary_file_1.docx]

**Supplementary Figure S1**

*Boxplots of Outcome Measures at Pre-training (T₁) and Three-Month Follow-Up (T₃) by Delivery Format*

**WEMWBS before and 3 months after training**


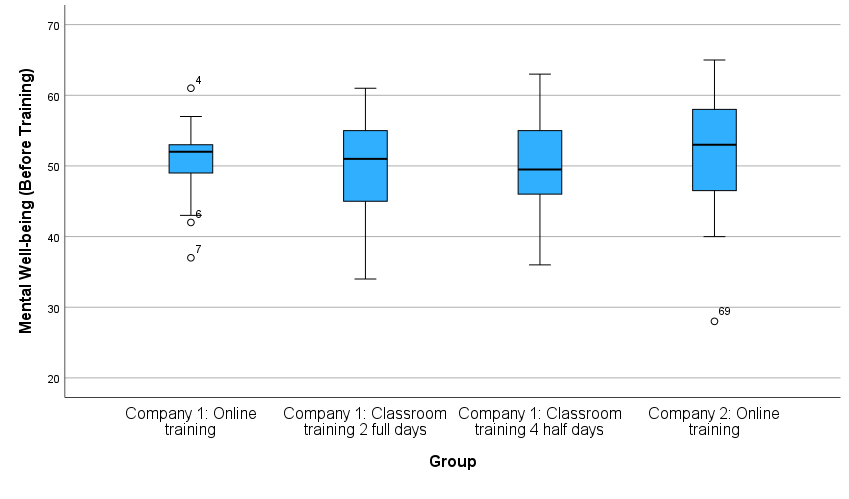


**Supplementary Table S1**

*Shapiro–Wilk Tests of Normality for Key Outcome Measures by Group and Time*

| Outcome | Time | Group | W | df | p-value | Normal? |
| --- | --- | --- | --- | --- | --- | --- |
| WEMWBS | T1 | Online training | 0.954 | 17 | 0.52 | Normal |
| WEMWBS | T1 | Classroom (2 × full days) | 0.945 | 19 | 0.32 | Normal |
| WEMWBS | T1 | Classroom (4 × half days) | 0.978 | 14 | 0.965 | Normal |
| WEMWBS | T3 | Online training | 0.964 | 17 | 0.704 | Normal |
| WEMWBS | T3 | Classroom (2 × full days) | 0.843 | 19 | 0.005 | Non-normal |
| WEMWBS | T3 | Classroom (4 × half days) | 0.927 | 14 | 0.279 | Normal |
| SWLS | T1 | Online training | 0.865 | 17 | 0.019 | Non-normal |
| SWLS | T1 | Classroom (2 × full days) | 0.936 | 19 | 0.225 | Normal |
| SWLS | T1 | Classroom (4 × half days) | 0.946 | 14 | 0.494 | Normal |
| SWLS | T3 | Online training | 0.837 | 17 | 0.007 | Non-normal |
| SWLS | T3 | Classroom (2 × full days) | 0.737 | 19 | 0.0 | Non-normal |
| SWLS | T3 | Classroom (4 × half days) | 0.963 | 14 | 0.779 | Normal |
| UWES | T1 | Online training | 0.915 | 17 | 0.124 | Normal |
| UWES | T1 | Classroom (2 × full days) | 0.875 | 19 | 0.017 | Non-normal |
| UWES | T1 | Classroom (4 × half days) | 0.938 | 14 | 0.399 | Normal |
| UWES | T3 | Online training | 0.802 | 17 | 0.002 | Non-normal |
| UWES | T3 | Classroom (2 × full days) | 0.929 | 19 | 0.166 | Normal |
| UWES | T3 | Classroom (4 × half days) | 0.825 | 14 | 0.01 | Non-normal |
| PSS | T1 | Online training | 0.869 | 17 | 0.021 | Non-normal |
| PSS | T1 | Classroom (2 × full days) | 0.889 | 19 | 0.031 | Non-normal |
| PSS | T1 | Classroom (4 × half days) | 0.954 | 14 | 0.629 | Normal |
| PSS | T3 | Online training | 0.942 | 17 | 0.337 | Normal |
| PSS | T3 | Classroom (2 × full days) | 0.913 | 19 | 0.085 | Normal |
| PSS | T3 | Classroom (4 × half days) | 0.917 | 14 | 0.197 | Normal |

**Supplementary Table S2**

*Median and Interquartile Range of Outcome Measures at Pre-training (T₁) and Follow-up (T₃) by Delivery Format*

| **Outcome** | **Time** | **Group** | **Median** | **IQR** |
| --- | --- | --- | --- | --- |
| **WEMWBS** | T1 | Online training | 52.0 | 6.0 |
| **WEMWBS** | T1 | Classroom (2 × full days) | 51.0 | 11.0 |
| **WEMWBS** | T1 | Classroom (4 × half days) | 49.5 | 10.0 |
| **WEMWBS** | T3 | Online training | 53.0 | 7.0 |
| **WEMWBS** | T3 | Classroom (2 × full days) | 55.0 | 10.0 |
| **WEMWBS** | T3 | Classroom (4 × half days) | 48.0 | 13.0 |
| **SWLS** | T1 | Online training | 26.0 | 5.0 |
| **SWLS** | T1 | Classroom (2 × full days) | 25.0 | 13.0 |
| **SWLS** | T1 | Classroom (4 × half days) | 21.5 | 11.0 |
| **SWLS** | T3 | Online training | 29.0 | 9.0 |
| **SWLS** | T3 | Classroom (2 × full days) | 27.0 | 5.0 |
| **SWLS** | T3 | Classroom (4 × half days) | 21.0 | 5.0 |
| **Flourish** | T1 | Online training | 48.0 | 4.0 |
| **Flourish** | T1 | Classroom (2 × full days) | 48.0 | 6.0 |
| **Flourish** | T1 | Classroom (4 × half days) | 46.0 | 8.0 |
| **Flourish** | T3 | Online training | 48.0 | 4.0 |
| **Flourish** | T3 | Classroom (2 × full days) | 48.0 | 7.0 |
| **Flourish** | T3 | Classroom (4 × half days) | 45.0 | 6.0 |
| **UWES** | T1 | Online training | 5.0 | 1.0 |
| **UWES** | T1 | Classroom (2 × full days) | 5.0 | 2.33 |
| **UWES** | T1 | Classroom (4 × half days) | 4.17 | 1.75 |
| **UWES** | T3 | Online training | 5.0 | 0.33 |
| **UWES** | T3 | Classroom (2 × full days) | 5.0 | 1.33 |
| **UWES** | T3 | Classroom (4 × half days) | 5.0 | 1.08 |
| **PSS** | T1 | Online training | 6.0 | 4.0 |
| **PSS** | T1 | Classroom (2 × full days) | 5.0 | 4.0 |
| **PSS** | T1 | Classroom (4 × half days) | 8.0 | 3.0 |
| **PSS** | T3 | Online training | 6.0 | 4.0 |
| **PSS** | T3 | Classroom (2 × full days) | 5.0 | 3.0 |
| **PSS** | T3 | Classroom (4 × half days) | 7.5 | 2.0 |

**Supplementary Table S3**

*Kruskal–Wallis Tests for Between-Group Differences in Outcome Measures at Pre-training (T₁) and Three-Month Follow-Up (T₃)*

| **Outcome** | **Time** | **H** | **df** | **p-value** | **Significant?** |
| --- | --- | --- | --- | --- | --- |
| **WEMWBS** | T1 | 0.302 | 2 | 0.86 | No |
| **WEMWBS** | T3 | 2.956 | 2 | 0.228 | No |
| **SWLS** | T1 | 4.022 | 2 | 0.134 | No |
| **SWLS** | T3 | 10.907 | 2 | 0.004 | Yes |
| **Flourish** | T1 | 2.391 | 2 | 0.303 | No |
| **Flourish** | T3 | 4.311 | 2 | 0.116 | No |
| **UWES** | T1 | 3.549 | 2 | 0.17 | No |
| **UWES** | T3 | 1.631 | 2 | 0.442 | No |
| **PSS** | T1 | 7.184 | 2 | 0.028 | Yes |
| **PSS** | T3 | 9.911 | 2 | 0.007 | Yes |

**Supplementary Table S4**

*Friedman Test Statistics for Within-Group Comparisons Across Pre-training (T₁), Midpoint (T₂) and Three-Month Follow-Up (T₃) by Outcome and Delivery Format*

| **Outcome** | **Group** | **Chi-square** | **df** | **p-value** | **Significant?** |
| --- | --- | --- | --- | --- | --- |
| **WEMWBS** | Online training | 10.308 | 2 | 0.006 | Yes |
| **WEMWBS** | Classroom (2 × full days) | 7.088 | 2 | 0.029 | Yes |
| **WEMWBS** | Classroom (4 × half days) | 1.024 | 2 | 0.599 | No |
| **SWLS** | Online training | 1.256 | 2 | 0.534 | No |
| **SWLS** | Classroom (2 × full days) | 3.169 | 2 | 0.205 | No |
| **SWLS** | Classroom (4 × half days) | 4.619 | 2 | 0.099 | No |
| **Flourish** | Online training | 3.436 | 2 | 0.179 | No |
| **Flourish** | Classroom (2 × full days) | 5.094 | 2 | 0.078 | No |
| **Flourish** | Classroom (4 × half days) | 4.333 | 2 | 0.115 | No |
| **UWES** | Online training | 6.056 | 2 | 0.048 | Yes |
| **UWES** | Classroom (2 × full days) | 13.855 | 2 | 0.001 | Yes |
| **UWES** | Classroom (4 × half days) | 11.2 | 2 | 0.004 | Yes |
| **PSS** | Online training | 1.676 | 2 | 0.433 | No |
| **PSS** | Classroom (2 × full days) | 3.825 | 2 | 0.148 | No |
| **PSS** | Classroom (4 × half days) | 0.389 | 2 | 0.823 | No |

**Supplementary Table S5**

*Wilcoxon Signed-Ranks Test Z Statistics, Sample Sizes and Rank-Biserial Effect Sizes (r) for Pre- to Three-Month Follow-Up Comparisons by Outcome and Group*

| **Outcome** | **Group** | **Z** | **N** | **Effect size (r)** |
| --- | --- | --- | --- | --- |
| **WEMWBS** | Online training | -2.19 | 17 | -0.531 |
| **SWLS** | Online training | -1.048 | 17 | -0.254 |
| **Flourish** | Online training | 0.0 | 17 | 0.0 |
| **UWES** | Online training | -0.546 | 17 | -0.132 |
| **PSS** | Online training | -0.078 | 17 | -0.019 |
| **WEMWBS** | Classroom (2 × full days) | -2.474 | 19 | -0.568 |
| **SWLS** | Classroom (2 × full days) | -1.923 | 19 | -0.441 |
| **Flourish** | Classroom (2 × full days) | -1.782 | 19 | -0.409 |
| **UWES** | Classroom (2 × full days) | -2.378 | 19 | -0.546 |
| **PSS** | Classroom (2 × full days) | -1.937 | 19 | -0.444 |
| **WEMWBS** | Classroom (4 × half days) | -0.312 | 14 | -0.083 |
| **SWLS** | Classroom (4 × half days) | -0.507 | 14 | -0.136 |
| **Flourish** | Classroom (4 × half days) | -1.109 | 14 | -0.296 |
| **UWES** | Classroom (4 × half days) | -1.699 | 14 | -0.454 |
| **PSS** | Classroom (4 × half days) | -0.829 | 14 | -0.222 |
